# Elevational changes in insect herbivory on woody plants in six mountain ranges of temperate Eurasia: Sources of variation

**DOI:** 10.1002/ece3.9468

**Published:** 2022-11-05

**Authors:** Mikhail V. Kozlov, Vitali Zverev, Elena L. Zvereva

**Affiliations:** ^1^ Department of Biology University of Turku Turku Finland

**Keywords:** elevational gradient, feeding guilds, insect herbivory, latitude, leaf functional traits, woody plants

## Abstract

Current theory predicts that the intensity of biotic interactions, particularly herbivory, decreases with increasing latitude and elevation. However, recent studies have revealed substantial variation in both the latitudinal and elevational patterns of herbivory. This variation is often attributed to differences in study design and the type of data collected by different researchers. Here, we used a similar sampling protocol along elevational gradients in six mountain ranges, located at different latitudes within temperate Eurasia, to uncover the sources of variation in elevational patterns in insect herbivory on woody plant leaves. We discovered a considerable variation in elevational patterns among different mountain ranges; nevertheless, herbivory generally decreased with increasing elevation at both the community‐wide and individual plant species levels. This decrease was mostly due to openly living defoliators, whereas no significant association was detected between herbivory and elevation among insects living within plant tissues (i.e., miners and gallers). The elevational decrease in herbivory was significant for deciduous plants but not for evergreen plants, and for tall plants but not for low‐stature plants. The community‐wide herbivory increased with increases in both specific leaf area and leaf size. The strength of the negative correlation between herbivory and elevation increased from lower to higher latitudes. We conclude that despite the predicted overall decrease with elevation, elevational gradients in herbivory demonstrate considerable variation, and this variation is mostly associated with herbivore feeding habits, some plant traits, and latitude of the mountain range.

## Graphical abstract

We used a standardized sampling protocol along elevational gradients in six mountain ranges, located at different latitudes within temperate Eurasia, to uncover the sources of variation in elevational patterns in insect herbivory on woody plant leaves at both community and species levels. We found that an overall decrease in herbivory with increasing elevation was mostly due to openly living defoliators, whereas no significant association was detected between herbivory and elevation among insects living within plant tissues (i.e., miners and gallers). The strength of the negative correlation between herbivory and elevation increased from lower to higher latitudes.

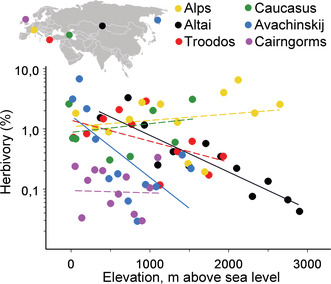

## 1 INTRODUCTION

Mountains, which cover 19%–24% of the global land area, are widely recognized for their economic, ecological, and social values (Sayre et al., 2018). Despite their fragility, mountain ecosystems host a substantial fraction of global biodiversity (Körner, 2004; Payne et al., 2020) and serve as its refugia through major eco‐climatic changes (Meng et al., 2019). Consequently, studies of biotic interactions in mountain regions are crucial for revealing the mechanisms that generate and maintain the high‐diversity mosaic of mountain communities in spatially and temporally variable environments (Chapin & Körner, 1995).

Since the time of Alexander von Humboldt (1817), mountains have served as natural laboratories for exploring ecological patterns and environmental processes, with a particular emphasis on the effects of abiotic factors on species diversity, trait evolution, biotic interactions, and ecosystem services at both the ecological and evolutionary time scales (Malhi et al., 2010; Tito et al., 2020). In this respect, mountain slopes offer some advantages over latitudinal gradients when studying biotic interactions along environmental gradients due to the short spatial distances between localities with different climates and productivities and due to the similar day lengths at different elevations (Körner, 2007).

Current theory predicts that the intensity of biotic interactions, particularly herbivory, decreases with increasing latitude and elevation (Pellissier et al., 2012; Schemske et al., 2009). However, recent studies have revealed great variations in the direction, strength, and shape of both latitudinal and elevational changes in herbivory (Moles et al., 2011; Moreira et al., 2018; Zvereva & Kozlov, 2021). Contradictions exist between studies describing environmental gradients in the strength of biotic interactions, and these discrepancies are often explained by differences in methodology, particularly differences in the study design and types of data collected by different researchers (Andrew et al., 2012; Anstett et al., 2016). These issues could be overcome by the use of standardized methodology for studying biotic interactions across geographic environmental gradients; therefore, not surprisingly, studies that have used this approach (e.g., Hargreaves et al., 2019; McKinnon et al., 2010; Roslin et al., 2017) have generated the most convincing and consistent patterns. However, elevational studies involving gradients replicated across large latitudinal ranges are almost completely lacking in the published literature (but see Hargreaves et al., 2019).

The species composition of both plants and herbivores changes considerably with elevation; therefore, the patterns observed in individual plant species may not reflect the elevational changes in the role of herbivory in mountain ecosystems (Zvereva et al., 2022). Nevertheless, most studies of elevational gradients in herbivory are conducted on a single plant species (reviewed by Moreira et al., 2018), whereas community‐wide studies are rare (but see De Long et al., 2016; Descombres et al., 2020; Martini et al., 2021; Metcalfe et al., 2014; Zvereva et al., 2022). Community‐wide estimates are essential for understanding the consequences of herbivory on plant community structure and ecosystem functioning (Anstett et al., 2016); consequently, comparisons between species‐specific and community‐wide approaches taken to quantify herbivory patterns within the same elevational gradient are critically required to learn whether the elevational patterns in herbivory observed for individual plant species can be extrapolated to the entire plant community.

The strength and shape of the elevational gradients in herbivory may depend on the geographic position of a given mountain range (Galmán et al., 2018; Zvereva et al., 2022), because changes in abiotic conditions with elevation may follow different patterns depending on climate and latitude of the mountain range. For example, the lapse rate (temperature decrease per unit elevation change) may depend on the latitude of a particular mountain area (Rolland, 2002; Wang et al., 2011). Also, the altitude of the treeline position, at which environmental conditions change abruptly, decreases with an increase in latitude (Körner, 1998; Paulsen & Körner, 2014). Therefore, the difference in ambient air temperature between forest and alpine habitats is lower at high latitudes than at low latitudes. Consequently, we predict that the association between herbivory and elevation is stronger at high latitudes, where the vegetation and microclimate change faster with elevation. The best test of this prediction would be to compare elevational changes in herbivory between mountain ranges located at different latitudes.

The majority of studies addressing elevational patterns in insect herbivory are focused on defoliating insects or they combine the damage imposed by several feeding guilds. By contrast, studies comparing elevational changes between herbivore feeding guilds (e.g., Garibaldi et al., 2011) are infrequent, despite reports that changes in herbivory along environmental gradients may vary among different feeding guilds (Andrew et al., 2012; Carmona et al., 2020). In particular, herbivores feeding inside plant tissues, in contrast to externally feeding insects, would be protected from the direct impacts of the abiotic environment (Price et al., 1987; Tooker & Giron, 2020); consequently, endophagous and exophagous herbivores may respond differently to environmental gradients. Notably, internal feeding would not protect insects from unfavorable thermal conditions, because the temperatures inside plant tissues follow the ambient air temperatures (Levitt, 1980; Price et al., 1987), but the microenvironment within plant tissues would successfully protect insects from desiccation (Tooker & Giron, 2020). The danger of desiccation for insects increases with elevation because of increasing solar radiation and wind (Körner, 2007); therefore, we predict a stronger decrease in plant damage due to openly living defoliators than to internally feeding miners and gallers with increasing elevation.

Plant species may exhibit different elevational changes in herbivory, and these differences are partly related to the plant growth form (woody vs. herbaceous) and leaf longevity (evergreen vs. deciduous) (Czwienczek, 2012; Galmán et al., 2018). For example, global analysis has revealed a decrease in herbivory with an increase in elevation in deciduous woody species, but not in herbaceous species or in evergreen woody plants (Galmán et al., 2018). Elevational patterns in herbivory may also differ between tall and low plants (Zvereva et al., 2022), because surface temperatures in alpine zones are considerably higher than air temperatures, whereas these temperatures show an opposite relationship in forest zones (Graae et al., 2012). The slower elevational decrease in surface temperatures relative to air temperatures may create favorable conditions for herbivores feeding on low‐stature plants in alpine habitats; therefore, decreases in herbivory with increasing elevation may be weaker in these plants than in tall plants.

Changes in the abiotic environment along elevational gradients also cause variations in functional leaf traits (Read et al., 2014). For example, leaf size and specific leaf area (SLA) generally decrease with increasing elevation (Midolo et al., 2019; Wright et al., 2017), whereas leaf water content tends to increase (Cruz‐Maldonado et al., 2021; Yang et al., 2021), despite the considerable variations in elevational patterns reported among individual studies. All these traits are associated with plant quality for herbivores (Callis‐Duehl et al., 2017; Wright et al., 2004), so their changes can contribute to elevational changes in herbivory. In the present study, we tested this hypothesis by recording leaf size, SLA, and leaf water content in plant individuals in which we measured herbivory.

We used a similar protocol to measure herbivory and plant traits along elevational gradients in six mountain ranges located at different latitudes within temperate Eurasia. Our goal was to uncover the sources of variation in elevational patterns in insect herbivory on woody plant leaves; therefore, we tested whether (i) herbivory generally decreases with an increase in elevation in studied mountain ranges; (ii) community‐wide herbivory and herbivory estimated on individual plant species follow similar elevational patterns; (iii) decreases with elevation are stronger for openly living defoliators than for miners and gallers feeding inside plant tissues; (iv) elevational changes in herbivory are stronger in mountain ranges located at higher latitudes; (v) elevational changes in herbivory depend on plant height (tall or low) or life‐history traits (deciduous or evergreen); and (vi) elevational patterns in herbivory are associated with elevational changes in leaf functional traits (size, SLA, and water content).

## 2 MATERIALS AND METHODS

### 2.1 Study regions and study sites

We selected six mountain ranges in Eurasia (Figure S1, Table 1 and Table S1) to maximize variations in latitude, vegetation types, and geology among the study regions. The lowest elevation sites were located in coniferous forests (Altai and Cairngorms), deciduous forests (Alps and Avachinskij), Mediterranean scrub (Troodos), and semi‐deserts (Caucasus). The highest elevation sites were located in mixed forest (Troodos), alpine meadows (Caucasus), and alpine tundra (all other regions).

**TABLE 1:** Study regions, collection dates, and sample sizes

Mountain range and country	Collection dates	Elevation, m		Latitude, °N	Numbers of			
		Min	Max		Sites	Plant species	Plant individuals	Leaves
Alps, France	21–30.ix.2017	60	2650	45	13	31	312	23,850
Altai, Russia	3–8.viii.2021	370	2900	51	13	56	237	30,208
Avachinskij, Russia	14–16.viii.2021	20	1520	53	12	27	197	17,605
Cairngorms, UK	1–8.viii.2018	60	1095	57	12	18	286	28,852
Caucasus, Russia	25.vi–3.vii.2021	−27	1540	43	8	48	110	9919
Troodos, Cyprus	12–18.x.2021	30	1930	35	11	44	150	21,904

Within each mountain range, our aim was to collect data from 10 to 14 study sites regularly spaced along an elevational gradient. We estimated the desired elevations for data collection before visiting the study region, and we then selected (often with the assistance of local scientists) the study site with the least disturbed plant community among several accessible sites located at the chosen elevation. We were unsuccessful in collecting the planned number of samples in the Caucasus because of the virtual absence of woody plants in the alpine meadows and the scarcity of natural vegetation at low elevations.

### 2.2 Sampling protocol

The sampling was conducted in the second half of the growth season, when the majority of insect herbivores had completed their feeding but well before the start of leaf fall at any of the study sites. Naturally, the growth period decreases with an increase in elevation; and this decrease could contribute to elevational changes in leaf damage. However, keeping in mind that the greatest part of the damage is imposed on young leaves (e.g., Aide, 1993; Coley, 1983), we assume that plant losses to insects in both low‐ and high‐elevation sites at the time of sampling approached their seasonal maximum and the confounding effect of phenology on our estimates of herbivory is therefore unlikely.

At each site, we collected a total of 15–25 branches (greater numbers corresponded to more diverse plant communities) from common woody plant species. We selected branches for sampling while standing at a distance of 5–10 m away (at this distance, the level of herbivory on the selected branch could not be assessed visually) to avoid unconscious selection bias. We disregarded a few branches that were grazed by vertebrate herbivores, which usually remove the entire shoots or greater parts of adjacent leaves; this ‘coarse’ damage is easily distinguished from a ‘fine’ and relatively minor damage imposed on individual leaves by invertebrate herbivores. All samples were collected and processed by the same persons (MVK and VZ) who had no a priori knowledge of the herbivory levels in the visited regions and/or the collected plant species.

In the Alps and in the Cairngorms, we first selected up to five woody plant species that dominated the community (i.e., that comprised the greater part of foliar biomass), and we then haphazardly collected one branch (or group of branches) from each of the five mature individuals of each selected species (on a first‐found, first‐sampled basis). In the other mountain ranges, we did not select the species to be collected. Instead, we collected one branch from each of 15–25 individuals of woody plants irrespective of their identity using the following procedure. Each of the two collectors walked along a straight line, making stops at approximately 10 m intervals and collecting a branch from a species, which had the greatest foliar biomass (as estimated visually) within a 2 × 10 m plot behind the collector. This change was introduced to better reflect herbivory in species‐rich plant communities and to avoid possible subjectivity in the selection of plant species to be collected. All other details of the protocol were the same across all regions, and we assumed that under both these protocols the numbers of sampled individuals of each species were roughly proportional to the contributions of these species to the community‐wide foliar biomass. Importantly, the sampling protocol was always identical within each gradient, and thus a minor difference outlined above is unlikely to influence the results of meta‐analysis based on correlations between herbivory and elevation within individual gradients.

### 2.3 Measurements of herbivory

In the laboratory, each leaf was carefully examined for the presence of damage imposed by chewing, galling, and mining invertebrates. In conifers, we (as in the previous study: Zvereva et al., 2020) classified current‐year needles that were missing from the shoot as having been consumed by insects, because undamaged needles of evergreen conifers generally persist for several years (Kozlov et al., 2009).

We assigned each leaf/needle (leaf hereafter) to one of the damage classes according to the percentage of the leaf area that was consumed or otherwise damaged by insects, as follows: 0 (intact leaves), 0.01%–1%, 1%–5%, 5%–25%, 25%–50%, 50%–75%, and 75%–100%. The last class included the petioles of fully consumed leaves and missing needles. We calculated the proportion of foliage area lost to insects (i.e., the leaf herbivory level) by multiplying the number of leaves in each damage class by the respective median values of the damaged leaf area (i.e., 0 for intact leaves, 0.5% for the damage class 0.01%–1%, etc.); the obtained values were then summed for all damage classes and divided by the total number of leaves (including undamaged ones) in a sample (Alliende, 1989; Kozlov et al., 2015; Zvereva et al., 2020).

### 2.4 Measurements of leaf traits

We measured leaf traits from each branch collected from Altai, Avachinskij, Caucasus, and Troodos, but only from one of several conspecific branches collected from the same site in the Alps and in the Cairngorms. Soon after branch collection, when the plant leaves were still turgid, we sampled 1–125 undamaged leaves (depending on leaf size; the median value was 8 leaves) and weighed them to the nearest 1 mg. We then punched out 5–12 disks (4.5, 8, or 12 mm in diameter, depending on leaf size) from those leaves, dried the disks and the remaining parts of leaves for 24 h at 105°C, and then weighed them to the nearest 0.1 mg.

When the leaves were too small to allow punching of leaf disks, we press‐dried the leaves and measured their total area from their photographs using Adobe Photoshop 2020. This measured area was then divided by 0.902 to correct for leaf shrinkage during drying. This correction factor was obtained by averaging the ratio between dry and fresh areas of 60 leaf disks collected from the leaves of 20 plant species from our study regions. We did not measure SLA in needle‐bearing plants. The dry weight of an individual leaf was calculated by dividing the weight of the punched leaves plus disks by the number of leaves. The water content in leaf tissues was calculated by dividing the difference between the wet and dry weight of a leaf by its wet weight. The SLA was calculated as the total area of the disks or leaves (mm^2^) divided by their dry weight (mg). Leaf area (mm^2^) was calculated by multiplying dry leaf weight (mg) by SLA (mm^2^ mg^−1^).

### 2.5 Data analysis

Our attempt to explore the proportion of insect damage in leaves using a generalized linear mixed model with a beta error distribution and a logit link function failed: the model based on 1289 sample‐specific values did not converge. Therefore, we used a log_10_(*x* + 0.01) transformation to normalize the distribution of the residuals, and we averaged the herbivory data (separately for defoliators, gallers, and miners) for either site‐specific values (for community‐level analysis) or by plant species by site combinations (for species‐level analysis). The latter analysis was restricted to measurements of herbivory on the same plant species conducted at least in four study sites within the same mountain range. We classified the collected plant species (i) as low stature (the height of mature plants is generally below 70 cm) or tall (above 70 cm) and (ii) as evergreen or deciduous.

Following an approach developed by Galmán et al. (2018), we first tested whether elevation predicts total community‐wide herbivory across all 69 sites combined; we used the Pearson correlation coefficient for this analysis. We then used ANCOVA (with elevation as a covariate) to analyze differences in elevational changes in total herbivory among the study regions (classificatory variable). Finally, we used a linear mixed model (SAS GLIMMIX procedure; SAS Institute, 2009) with a Gaussian error distribution to compare the elevational patterns among feeding guilds. In this model, the region, feeding guild, elevation, and their interactions were considered fixed effects, whereas the study site nested within a region was treated as a random effect. We adjusted the standard errors and denominator degrees of freedom following Kenward and Roger (2009) and evaluated the significance of a random factor by testing the likelihood ratio against the *χ* ^2^ distribution (Littell et al., 2006).

To further evaluate sources of variation in elevational changes, we conducted a meta‐analysis. We quantified the strength of the elevational gradients by the *z*‐transformed Pearson correlation coefficients between elevation, herbivory, and leaf traits (*z*_r_). We compared *z*_r_ between regions, herbivore feeding guilds, groups of plants that differ in leaf longevity and stature or between community‐wide and species‐specific effects by calculating the between‐group heterogeneity (*Q*_B_) using a random effects meta‐analytical model, and we tested *Q*_B_ against the *χ* ^2^ distribution with the number of groups minus one degree of freedom. We then calculated an average value of *z*_r_ and its 95% confidence interval (CI_95_). The effects were considered significant when this confidence interval did not include zero. The association between the strength of the elevational gradients and the latitude of a study region was explored using meta‐regression (Koricheva et al., 2013).

## 3 RESULTS

### 3.1 Overview of the data

We examined 132,176 leaves collected from 1289 individuals of 177 plant species (Table 1). Among these, 22,785 leaves (17.2%) were damaged by invertebrates (primarily insects), as indicated by the size and shape of the consumed parts of the leaves and by traces of insect mandibulae on wound margins. We found 687 leaves with galls and 1065 leaves with mines.

Across the 69 study sites, plants lost (mean ± SE) 2.24 ± 0.23% of their leaf area to externally feeding defoliators, 0.036 ± 0.012% to gallers, and 0.111 ± 0.018% to miners; the total losses to insects amounted 2.38 ± 0.24%. The total herbivory at the lowest elevation site of each gradient did not correlate with the latitude of the study site (*r* = .02, *n* = 6 sites, *p* = .98).

### 3.2 Elevational changes in herbivory

**FIGURE 1:**
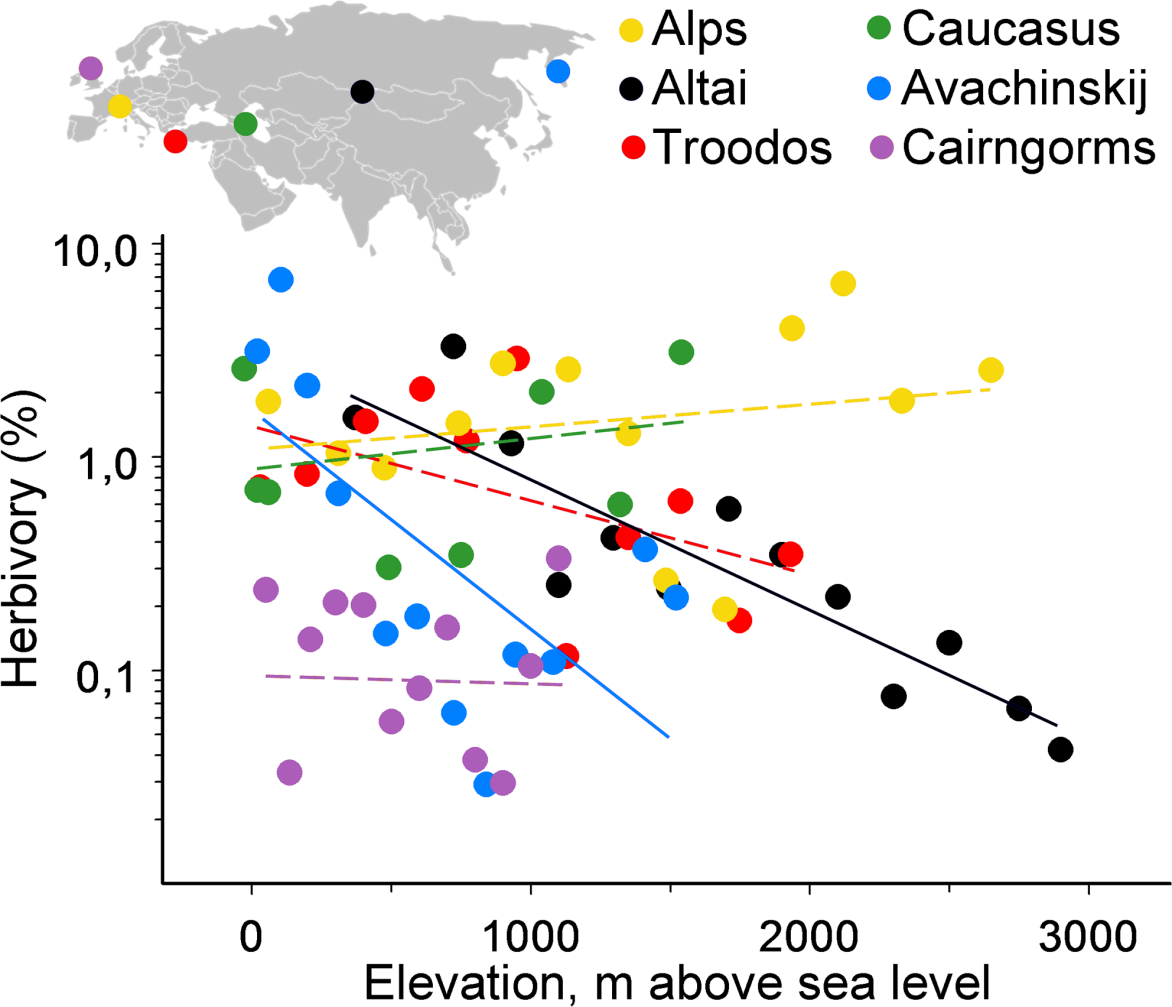
Elevational changes in total insect herbivory. The colors of both the data points and the approximating functions refer to mountain ranges; their positions within Eurasia are indicated on the insert above the graph. Solid lines: significant (*p* < .05) models; dashed lines: not statistically significant models.

The elevational patterns in total community‐wide herbivory differed among the six mountain ranges (ANCOVA, Region × Elevation interaction: *F*_5, 57_ = 4.15, *p* = .0028). The total herbivory significantly decreased with an increase in elevation in Altai and Avachinskij, but neither the decrease observed in Cairngorms and Troodos nor the increase observed in the Alps and the Caucasus was statistically significant (Figure 1). The analysis of the pooled data found no relationship between elevation and herbivory (*r* = .13, *n* = 69 sites, *p* = .29). By contrast, a meta‐analysis of gradient‐specific data (Tables S2 and S3) revealed a negative correlation between the total insect herbivory and elevation, and this did not differ between the community‐wide and species‐specific estimates (Figure 3; *Q*_B_ = 0.002, df = 1, *p* = .96).

**FIGURE 2:**
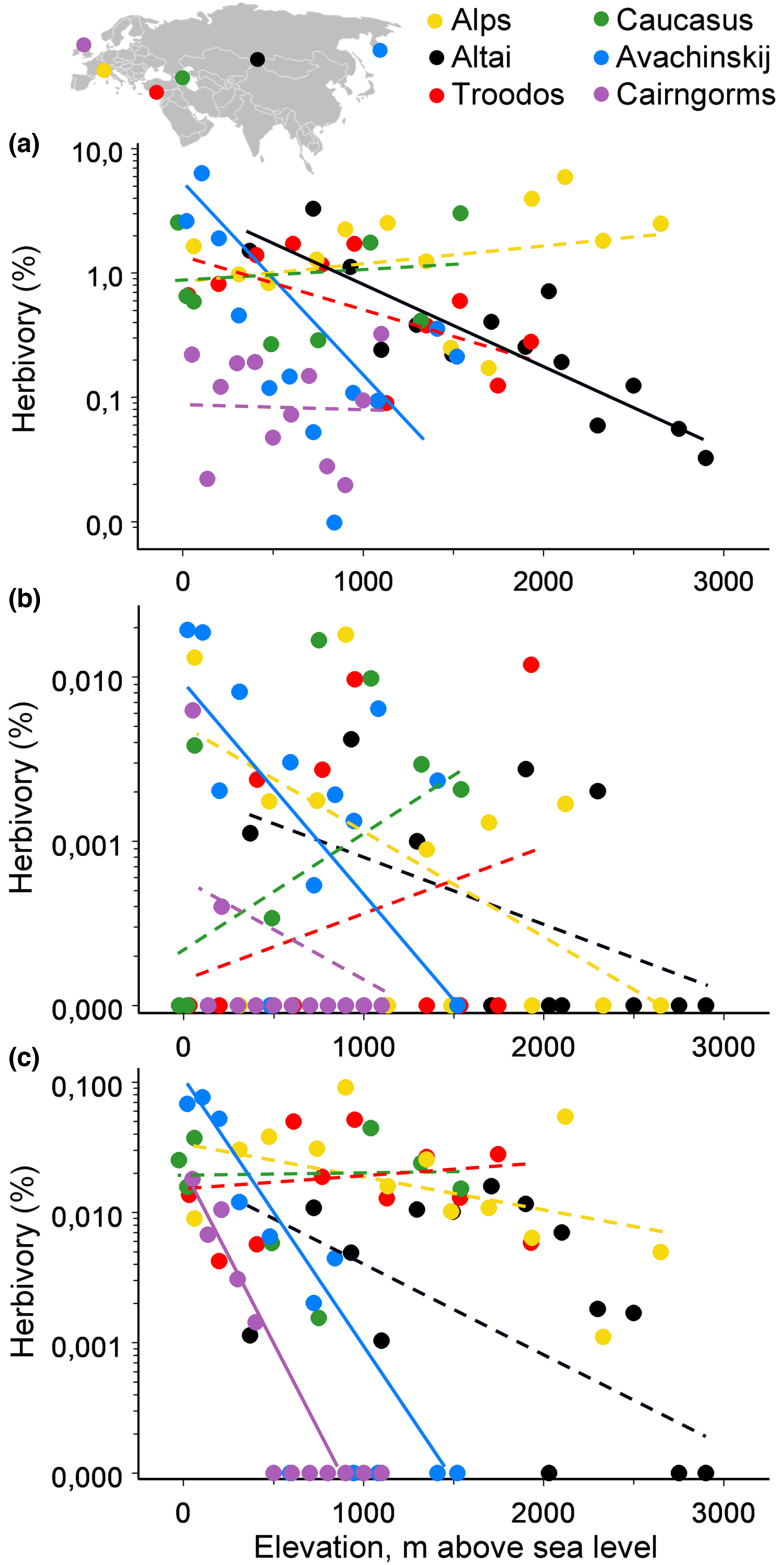
Elevational changes in guild‐specific herbivory: (a) defoliators; (b) gallers; (c) miners. The colors of both the data points and the approximating functions refer to mountain ranges; their positions within Eurasia are indicated on an insert above the graphs. Solid lines: significant (*p* < .05) models; dashed lines: non‐significant models.

The variation in elevational patterns among feeding guilds differed among mountain ranges (Table S4). Consistently, only five of the 18 guild‐by‐region data sets demonstrated any significant linear elevational patterns in community‐wide herbivory (Figure 2 and Table S2). The Avachinskij volcano was the only study region in which damage caused by all three herbivore feeding guilds decreased significantly with increasing elevation (Figure 2).

### 3.3 Factors explaining variation in elevational patterns in herbivory

**FIGURE 3:**
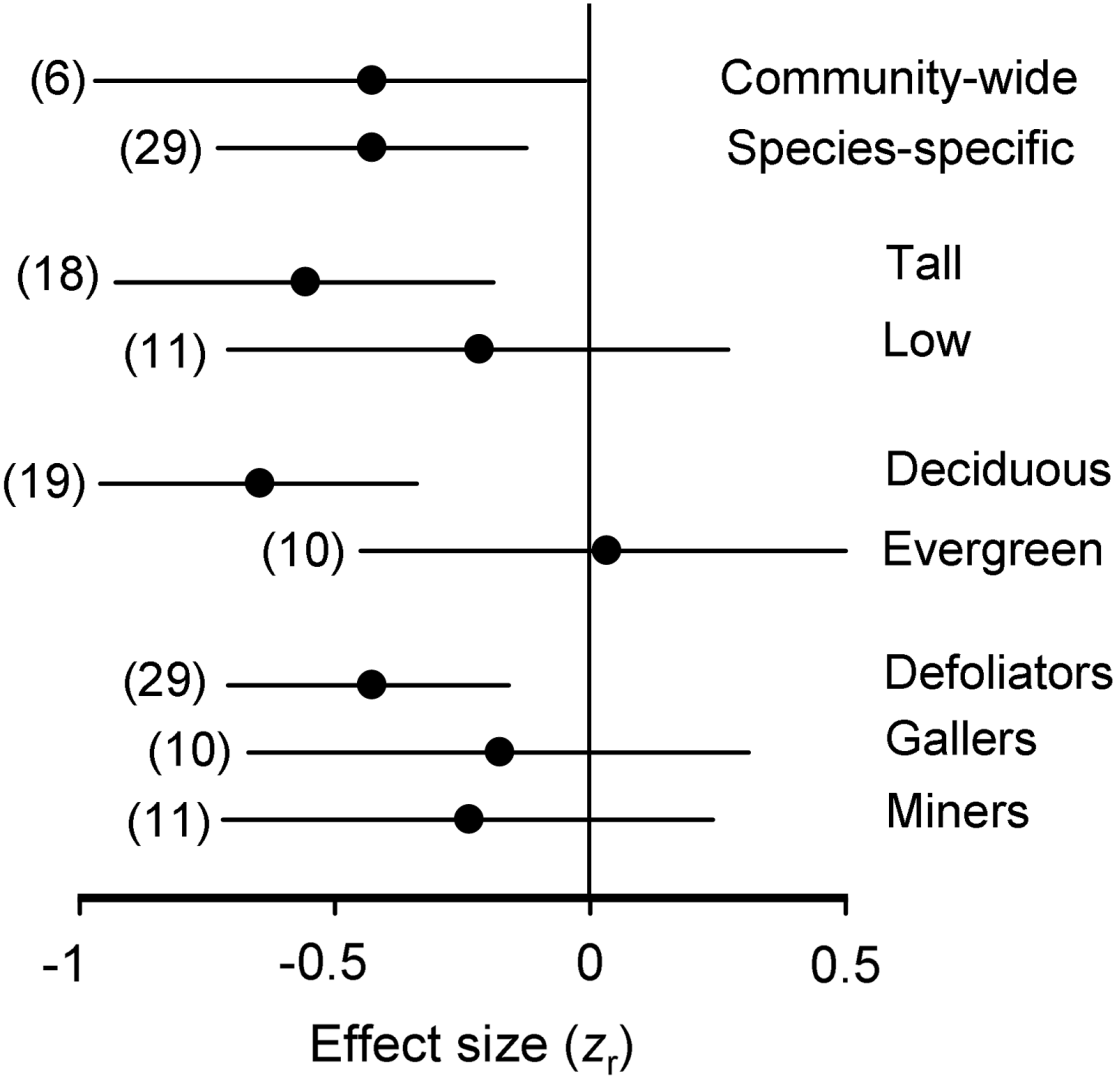
The strength of elevational changes in herbivory (*z*_r_) at the community‐wide level across mountain ranges and at the species level within these mountain ranges is shown overall and by explanatory variables. The negative effect size indicates a decrease in herbivory with an increase in elevation. Horizontal lines denote 95% confidence intervals; sample sizes are shown in parentheses.

The species‐specific herbivory on tall woody plants (trees and tall shrubs) significantly decreased with an increase in elevation, whereas the elevational changes in herbivory were not significant for the low shrubs (Figure 3; between‐group difference: *Q*_B_ = 1.44, df = 1, *p* = .22). Herbivory on deciduous plants decreased with elevation, while herbivory on evergreen plants did not show any elevational changes (Figure 3; between‐group difference: *Q*_B_ = 7.05, df = 1, *p* = .008). Plant losses to defoliators significantly decreased with increasing elevation, while the association between herbivory and elevation for gallers and miners was not significant (Figure 3).

**FIGURE 4:**
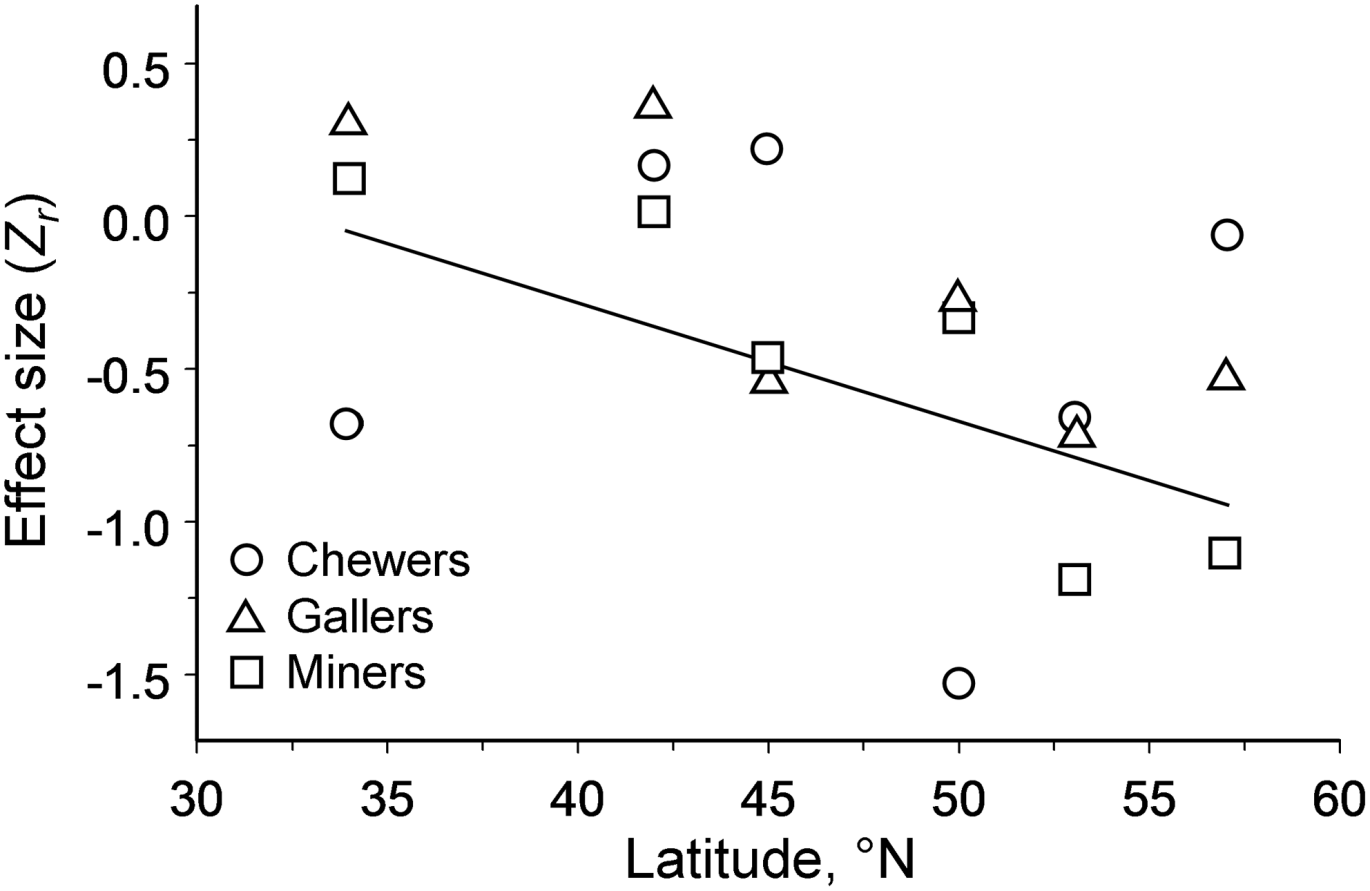
The strength of community‐wide elevational changes in herbivory (*z*_r_) averaged across three feeding guilds in relation to the latitude of each of six mountain regions (metaregression: *Q* = 5.64, df = 1, *p* = .013).

The strength of the elevational gradient in community‐wide herbivory (all guilds combined) did not depend on the difference in elevation between the highest and the lowest sites (*Q* = 0.36, df = 1, *p* = .28); rather, it became weaker with a decrease in latitudes of the study region (Figure 4).

Neither the community‐wide SLA nor water content changed with elevation (Table S5; *z*_r_ = −0.08, CI_95_ = −0.42…0.34 and *z*_r_ = 0.10, CI_95_ = −0.34…0.48, respectively, *n* = 6 regions). By contrast, leaf area of leaf‐bearing plants decreased with increasing elevation across six regions (*z*_r_ = −0.87, CI_95_ = −1.11…−0.61). The elevational changes in community‐wide herbivory were not associated with leaf water content (Table S6; *z*_r_ = −0.23, CI_95_ = −0.64…0.17) but increased with an increase in both SLA (*z*_r_ = 0.09, CI_95_ = 0.01…0.19) and leaf area (*z*_r_ = 0.42, CI_95_ = 0.17…0.67).

## 4 DISCUSSION

### 4.1 General pattern emerging from diverse responses

We have provided the first demonstration of the existence of considerable variations in elevational changes in herbivory among multiple mountain ranges. Nevertheless, these diverse responses yielded a general pattern: our meta‐analysis of gradient‐specific correlations showed an overall decrease in total herbivory on woody plant leaves with increasing elevation. This result, which is in line with the theoretical predictions (Carmona et al., 2020; Moreira et al., 2018; Sundqvist et al., 2013), supports our hypothesis (i).

### 4.2 Herbivore feeding guilds

The three insect herbivore feeding guilds studied by us demonstrated variable relationships with elevation (Figure 2). In support of our prediction (iii), and in line with previous findings (Sohn et al., 2019), the association with elevation was twofold stronger for openly living defoliators than for insects feeding inside plant tissues (miners and gallers). These results are also in line with the global study of elevational patterns in parasitism, where decreases with increasing elevation are greater for ectoparasitoids and parasitoids of ectophagous insects than for endoparasitoids and parasitoids of endophagous insects (Péré et al., 2013). All these findings jointly indicate that a concealed life habit makes insects less susceptible to the adverse and extreme climatic conditions that are typical of high elevations. The lack of between‐guild differences in elevational patterns reported by Garibaldi et al. (2011) may be explained by the location of their entire gradient within a forest zone, while five of the six gradients studied by us crossed the tree line and extended to the alpine zone, where the impacts of environmental factors detrimental to insects (e.g., irradiation and wind) are especially strong (Körner, 2007).

Despite the variation in the strength of the association with elevation among feeding guilds, the overall plant losses to herbivory follow the pattern created by defoliators—namely, a significant decrease with an increase in elevation—because internally feeding insects contribute only 6% to the total losses of foliage. Therefore, our further discussion addresses only the total plant losses to insect herbivores.

### 4.3 Herbivory and plant traits

The community‐wide herbivory averaged across six gradients and the total herbivory averaged across 29 plant species from these gradients show similar decreases with elevation (Figure 3), thus supporting our hypothesis (ii). This similarity is in line with the previously demonstrated consistency between intra‐ and interspecific variation in leaf traits along environmental gradients (Hulshof et al., 2013; Read et al., 2014).

The variation in plant traits is one of the factors driving spatial variation in insect herbivory (Defossez et al., 2018; Kozlov et al., 2015), and the leaf traits considered in our study are tightly linked to plant quality for herbivores. In particular, the SLA and water content are associated with mechanical and physical defense (Callis‐Duehl et al., 2017; Wright et al., 2004). Consistently, SLA in our gradients weakly but positively correlated with the total herbivory. Meta‐analysis revealed that SLA generally decreases with elevation, but this is mostly due to patterns observed in herbaceous plants, whereas SLA did not change with elevation in woody plants (Midolo et al., 2019). Our results are in line with the latter pattern, as we did not find any systematic changes in SLA across our elevational gradients. Nevertheless, the elevational pattern in insect herbivory discovered in our study is associated with both SLA and (presumably) with other traits of the leaf economic spectrum that highly correlate with SLA (Wright et al., 2004).

The leaf water content in our study plants does not change with elevation, and the patterns in leaf water content observed in our gradients are not associated with herbivory. Leaf area is the only trait that consistently decreases in our plants with an increase in elevation, in line with Nichlos et al. (2019), who explained this decrease using the leaf energy balance theory. The decrease in leaf area was consistent for the community‐wide and species‐specific values, in line with the conclusion by Read et al. (2014) that the abiotic environment in high‐elevation sites imposes selection for both small‐leaved genotypes and small‐leaved species. Small leaves are suggested to be less vulnerable to invertebrate herbivory because of their short expansion times (Moles & Westoby, 2000), and they are a low‐quality resource for mining and tying insects (Low et al., 2009; Marquis et al., 2002). Consistently, in our study, herbivory increased weakly but significantly with the increase in leaf size. We conclude that the contribution of the studied leaf traits to the elevational changes in herbivory along our gradients is minor, thus giving only partial support to our hypothesis (vi).

By contrast, the elevational patterns in herbivory are tightly associated with the life‐history traits of the study plants across our regions. Notably, herbivory decreases with increasing elevation on deciduous woody plants, but not on evergreen woody plants, and on tall but not on low‐stature woody plants. Different patterns of herbivory on deciduous and evergreen species have been previously found in both latitudinal (Zvereva et al., 2020) and elevational (Galmán et al., 2018; Zvereva et al., 2022) gradients. We suggest that the different responses of woody angiosperms and gymnosperms (comprising half of our evergreen species) to temperature changes (Zvereva & Kozlov, 2006) may have contributed to this difference. Another reason behind the lack of any pattern in evergreen plants is that herbivore damage is fivefold to sevenfold lower in gymnosperms than in angiosperms (Turcotte et al., 2014; Zvereva et al., 2020). In fact, the levels of herbivory in gymnosperms are so low that the measurement errors may exceed the average values of herbivory, thereby hampering the detection of any pattern (Kozlov & Zvereva, 2017).

Herbivory across our mountain ranges showed significant decreases with elevation on tall trees and shrubs but not on low‐stature woody plants (such as dwarf shrubs). The similar difference between tall and low woody plants observed in polar mountains (Zvereva et al., 2022) was explained by differences in the elevational gradients of air and near‐surface temperatures. In open alpine habitats, which represent the uppermost sites in five of six of our gradients, the soil surface temperatures are usually higher than the air temperatures due to the high solar irradiation (Graae et al., 2012; Körner, 1998). This creates a shallower temperature gradient experienced by the low‐stature plants growing near the soil surface than the air temperature gradient experienced by the canopies of trees and tall shrubs. The elevational patterns in herbivory are, to a great extent, shaped by temperature gradients; therefore, the association between herbivory and elevation is weaker in low‐stature plants than in tall plants. This difference between near‐surface and air temperatures may explain why previous global analysis (Galmán et al., 2018) discovered elevational changes in herbivory in woody plants (mostly tall trees and shrubs) but not in herbaceous (mostly low‐stature) plants. Furthermore, the elevational changes in the relative apparency of tall and low plants due to changes in vegetation structure could also contribute to the differences in elevational patterns observed in herbivory on tall and low plants.

Our results on the importance of plant life‐history traits for shaping elevational changes in herbivory, which confirm prediction (v), are in line with meta‐analysis, which found that plant life‐history traits are better predictors of plant susceptibility to chewing herbivores than are primary and secondary chemistry or physical leaf traits (Carmona et al., 2011).

### 4.4 Latitude and climate

The geographic variation in elevational patterns in biotic interactions has thus far been studied by two methods: (i) analyzing data from study sites located at different altitudes and latitudes but not forming continuous elevational gradients (Galmán et al., 2018; Roslin et al., 2017) and (ii) comparing patterns from continuous elevational gradients located at different latitudes (Hargreaves et al., 2019). We analyzed our data using both methods and found that the two methods yielded different results. Our data processed by the first method (i.e., by the correlation analysis of the pooled site‐specific data) did not reveal any significant elevational changes in community‐wide herbivory. By contrast, elevational changes became evident when using the second method (i.e., meta‐analysis of correlation coefficients between herbivory and elevation within individual mountain ranges). This difference clearly demonstrated the advantages of gradient studies versus analysis of scattered data because meta‐analysis of gradient studies could not be affected by absolute levels of herbivory.

Earlier studies hypothesized that the strength and shape of elevational gradients depend on the latitude of the mountain range (Galmán et al., 2018; Zvereva et al., 2022). In particular, the elevation gradients in herbivory were predicted to be steeper in tropics than in temperate regions due to the greater magnitude of change in climatic conditions from low to high elevations in the tropics relative to the temperate zone (Janzen, 1967), and because herbivory at the lower end of the elevation gradient is greater in the tropics than in the temperate regions (Galmán et al., 2018). The failure by Galmán et al. (2018) to detect any difference in elevational changes in herbivory between tropical and temperate regions may be explained (at least in part) by the use of data collected by different methods and by the application of the analysis, which does not account for variations among individual gradients. Our study, by analyzing data collected in several gradients, is the first to show that the strength of the negative correlation between herbivory and elevation increases from lower to higher latitudes within a temperate climate zone; that is, we discovered a pattern predicted by hypothesis (iv).

We conclude that elevational gradients in herbivory demonstrate considerable variation among mountain ranges, and that this variation is mostly associated with herbivore feeding habits, some plant traits, and latitude of the mountain range. The effect of latitude on elevational patterns in biotic interactions requires further investigation across a wider range of mountain regions located in different climates, from tropical to polar.

## AUTHOR CONTRIBUTIONS

**Mikhail V. Kozlov:** Conceptualization (equal); data curation (equal); formal analysis (equal); investigation (equal); methodology (equal); writing – original draft (equal); writing – review and editing (equal). **Vitali Zverev:** Investigation (equal); methodology (equal); visualization (equal); writing – review and editing (equal). **Elena L. Zvereva:** Conceptualization (equal); writing – original draft (equal); writing – review and editing (equal).

## CONFLICT OF INTEREST

The authors do not have any conflict of interest.

## Supporting information


Appendix S1

## Data Availability

All data are archived in the Dryad Digital Repository at https://doi.org/10.5061/dryad.j9kd51cgt.
