# Supplementary material for: Elevational changes in insect herbivory on woody plants in six mountain ranges of temperate Eurasia: Sources of variation
Source: Ecol Evol. 2022 Nov 5;12(11):e9468. doi: 10.1002/ece3.9468 (PMC9636509; doi:10.1002/ece3.9468)
Supplement: Supplementary file 1 — Appendix S1 [file ECE3-12-e9468-s001.doc]

**Elevational changes in insect herbivory on woody plants in six mountain ranges of temperate Eurasia: sources of variation**

Mikhail V. Kozlov*, Vitali Zverev, Elena L. Zvereva

Department of Biology, University of Turku, 20014 Turku, Finland

*Corresponding author: mikoz@utu.fi

ORCID

*Elena L. Zvereva* https://orcid.org/0000-0003-2934-3421

*Vitali Zverev* https://orcid.org/0000-0002-8090-9235

*Mikhail V. Kozlov* https://orcid.org/0000-0002-9500-4244

**Supplementary information**


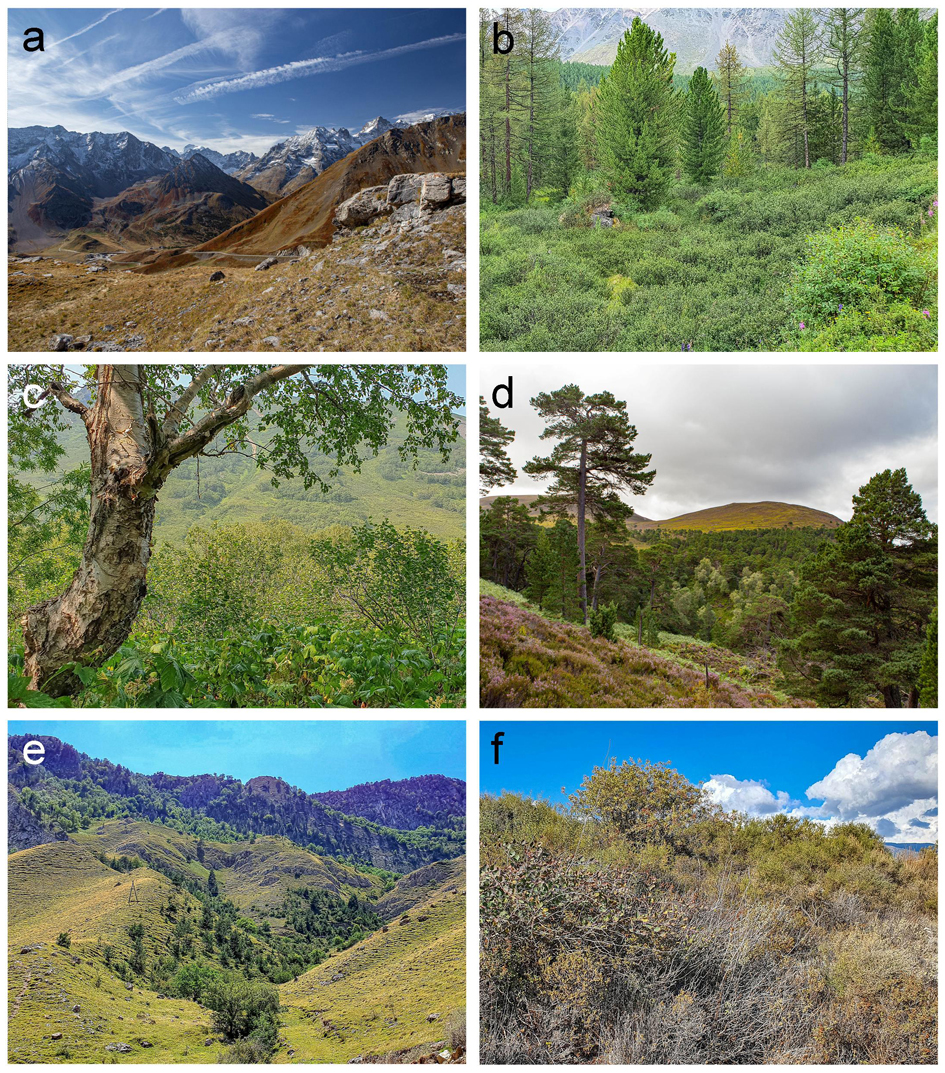
Figure S1. Landscapes at the study sites: a, Alps, France, 2650 m above sea level (a.s.l.); b, Altai, Russia, 1900 m a.s.l.; c, Avachinskij, Russia, 310 m a.s.l.; d, Cairngorms, United Kingdom, 400 m a.s.l.; e, Caucasus, Russia, 1000 m a.s.l.; f, Troodos, Cyprus, 770 m a.s.l.

Table S1. Coordinates of study sites.

| Mountain range | Latitude | Longitude | Elevation |
| --- | --- | --- | --- |
| Alps, France | 43º 36′ 31″ N | 7º 04′ 56″ E | 60 |
|  | 45º 03′ 02″ N | 5º 47′ 13″ E | 310 |
|  | 45º 03′ 27″ N | 5º 45′ 13″ E | 475 |
|  | 45º 02′ 07″ N | 6º 03′ 57″ E | 740 |
|  | 45º 12′ 26″ N | 6º 28′ 11″ E | 900 |
|  | 45º 02′ 18″ N | 6º 12′ 40″ E | 1135 |
|  | 45º 02′ 29″ N | 6º 15′ 42″ E | 1350 |
|  | 45º 10′ 01″ N | 6º 26′ 08″ E | 1485 |
|  | 45º 01′ 48″ N | 6º 21′ 50″ E | 1695 |
|  | 45º 05′ 40″ N | 6º 25′ 52″ E | 1935 |
|  | 45º 05′ 13″ N | 6º 25′ 53″ E | 2120 |
|  | 45º 03′ 03″ N | 6º 23′ 29″ E | 2330 |
|  | 45º 00′ 58″ N | 6º 23′ 25″ E | 2650 |
| Altai, Russia | 51º 37′ 24″ N | 85º 42′ 34″ E | 370 |
|  | 50º 43′ 25″ N | 86º 15′ 46″ E | 723 |
|  | 51º 14′ 27″ N | 85º 38′ 53″ E | 930 |
|  | 50º 21′ 05″ N | 87º 23′ 49″ E | 1100 |
|  | 50º 59′ 21″ N | 85º 40′ 44″ E | 1296 |
|  | 51º 03′ 55″ N | 85º 35′ 24″ E | 1494 |
|  | 50º 07′ 02″ N | 88º 20′ 44″ E | 1710 |
|  | 50º 30′ 23″ N | 87º 40′ 25″ E | 1900 |
|  | 50º 06′ 30″ N | 87º 48′ 10″ E | 2100 |
|  | 50º 04′ 10″ N | 87º 46′ 10″ E | 2300 |
|  | 50º 04′ 17″ N | 87º 44′ 34″ E | 2500 |
|  | 50º 19′ 53″ N | 87º 44′ 34″ E | 2750 |
|  | 50º 04′ 44″ N | 87º 43′ 31″ E | 2900 |
| Avachinskij, Russia | 53º 00′ 58″ N | 158º 50′ 56″ E | 20 |
|  | 52º 50′ 23″ N | 158º 08′ 47″ E | 105 |
|  | 53º 04′ 31″ N | 158º 38′ 18″ E | 199 |
|  | 53º 07′ 14″ N | 158º 37′ 55″ E | 310 |
|  | 53º 13′ 23″ N | 158º 40′ 25″ E | 480 |
|  | 53º 14′ 17″ N | 158º 41′ 31″ E | 592 |
|  | 53º 14′ 55″ N | 158º 43′ 03″ E | 724 |
|  | 53º 15′ 31″ N | 158º 44′ 20″ E | 840 |
|  | 53º 15′ 57″ N | 158º 44′ 32″ E | 945 |
|  | 53º 16′ 27″ N | 158º 44′ 37″ E | 1080 |
|  | 53º 16′ 13″ N | 158º 46′ 58″ E | 1410 |
|  | 53º 16′ 04″ N | 158º 47′ 17″ E | 1520 |
| Cairngorms, UK | 57º 26′ 04″ N | 4º 16′ 01″ W | 60 |
|  | 57º 26′ 37″ N | 4º 15′ 49″ W | 125 |
|  | 57º 23′ 56″ N | 4º 09′ 46″ W | 200 |
|  | 57º 06′ 55″ N | 3º 51′ 44″ W | 300 |
|  | 57º 06′ 59″ N | 3º 50′ 42″ W | 410 |
|  | 57º 06′ 47″ N | 3º 50′ 22″ W | 495 |
|  | 57º 06′ 50″ N | 3º 50′ 09″ W | 595 |
|  | 57º 05′ 42″ N | 3º 49′ 17″ W | 690 |
|  | 57º 05′ 21″ N | 3º 49′ 07″ W | 800 |
|  | 57º 05′ 12″ N | 3º 48′ 49″ W | 905 |
|  | 57º 05′ 03″ N | 3º 48′ 32″ W | 1010 |
|  | 57º 04′ 50″ N | 3º 48′ 30″ W | 1095 |
| Caucasus, Russia | 43º 17′ 32″ N | 47º 27′ 35″ E | -27 |
|  | 41º 50′ 39″ N | 48º 29′ 13″ E | 20 |
|  | 43º 00′ 23″ N | 47º 14′ 50″ E | 60 |
|  | 42º 43′ 38″ N | 46º 48′ 50″ E | 490 |
|  | 42º 28′ 11″ N | 46º 50′ 50″ E | 750 |
|  | 42º 46′ 28″ N | 46º 56′ 58″ E | 1040 |
|  | 42º 05′ 52″ N | 47º 27′ 47″ E | 1320 |
|  | 42º 05′ 19″ N | 47º 29′ 23″ E | 1540 |
| Troodos, Cyprus | 34º 55′ 07″ N | 32º 20′ 14″ E | 30 |
|  | 34º 42′ 37″ N | 32º 46′ 01″ E | 198 |
|  | 34º 44′ 05″ N | 32º 46′ 46″ E | 408 |
|  | 34º 48′ 37″ N | 32º 47′ 22″ E | 610 |
|  | 34º 49′ 54″ N | 32º 48′ 23″ E | 770 |
|  | 34º 52′ 50″ N | 32º 50′ 43″ E | 950 |
|  | 34º 53′ 15″ N | 32º 51′ 23″ E | 1128 |
|  | 34º 53′ 51″ N | 32º 51′ 13″ E | 1350 |
|  | 34º 54′ 28″ N | 32º 51′ 57″ E | 1536 |
|  | 34º 55′ 35″ N | 32º 52′ 44″ E | 1747 |
|  | 34º 56′ 08″ N | 32º 51′ 44″ E | 1930 |

Table S2. Pearson correlation coefficients (*r*) between log_10_(x+0.01)-transformed total community-wide loss of leaf area to insects and elevation within individual mountain ranges.

| Mountain range | Total | | Defoliators | | Gallers | | Miners | |
| --- | --- | --- | --- | --- | --- | --- | --- | --- |
|  | *r* | *P* | *r* | *P* | *r* | *P* | *r* | *P* |
| Alps | 0.194 | 0.53 | 0.221 | 0.47 | -0.476 | 0.10 | -0.434 | 0.14 |
| Altai | -0.901 | <.0001 | -0.913 | <.0001 | -0.259 | 0.39 | -0.323 | 0.28 |
| Avachinskij | -0.623 | 0.03 | -0.584 | 0.05 | -0.614 | 0.03 | -0.827 | 0.0009 |
| Cairngorms | -0.083 | 0.80 | -0.062 | 0.84 | -0.480 | 0.12 | -0.804 | 0.0016 |
| Caucasus | 0.212 | 0.61 | 0.157 | 0.71 | 0.354 | 0.42 | 0.014 | 0.91 |
| Troodos | -0.541 | 0.09 | -0.585 | 0.06 | 0.312 | 0.35 | 0.117 | 0.74 |

Table S3. Pearson correlation coefficients (*r*) between log_10_(x+0.01)-transformed loss of leaf area to insects (total and by feeding guilds) and elevation within individual plant species.

| Region | Plant species | Life form | Plant height | Sample size | Total | Defoliators | Gallers | Miners |
| --- | --- | --- | --- | --- | --- | --- | --- | --- |
| Alps | *Betula pendula* | Deciduous | Tall | 7 | 0.0227 | -0.1442 | 0.0033 | 0.6716 |
|  | *Hippophae rhamnoides* | . | Tall | 5 | -0.7956 | -0.7973 | 0.6347 | . |
|  | *Larix decidua* | Deciduous | Tall | 4 | -0.6891 | -0.6892 | . | . |
|  | *Vaccinium myrtillus* | Deciduous | Low | 4 | -0.8191 | -0.8444 | . | 0.8504 |
|  | COMMUNITY-WIDE | . | . | 13 | 0.1940 | 0.2207 | -0.4763 | -0.4342 |
| Altai | *Betula pendula* | Deciduous | Tall | 7 | -0.8238 | -0.8363 | 0.0220 | -0.1720 |
|  | *Caragana arborescens* | Deciduous | Tall | 4 | -0.6457 | -0.8996 | . | 0.9319 |
|  | *Larix sibirica* | Deciduous | Tall | 9 | 0.1430 | 0.1500 | . | . |
|  | *Lonicera caerulea* ssp. *altaica* | Deciduous | Tall | 5 | -0.8890 | -0.9324 | . | 0.2916 |
|  | *Picea obovata* | Evergreen | Tall | 4 | -0.2648 | -0.2647 | . | . |
|  | *Pinus sibirica* | Evergreen | Tall | 6 | -0.2110 | -0.2110 | . | . |
|  | *Salix saposhnikovii* | Deciduous | Tall | 5 | -0.7555 | -0.7319 | 0.0269 | . |
|  | COMMUNITY-WIDE | . | . | 13 | -0.9015 | -0.9125 | -0.2590 | -0.3231 |
| Avachinskij | *Alnus alnobetula* ssp. *fruticosa* | Deciduous | Tall | 6 | -0.6268 | -0.6602 | -0.5610 | -0.5688 |
|  | *Betula ermanii* | Deciduous | Tall | 6 | -0.9145 | -0.8956 | -0.4378 | -0.7742 |
|  | *Empetrum nigrum* | Evergreen | Low | 4 | 0 | 0 | . | . |
|  | *Kalmia procumbens* | Evergreen | Tall | 4 | 0.8035 | 0.8035 | . | . |
|  | *Salix arctica* | Deciduous | Low | 7 | 0.0369 | 0.0351 | 0.3207 | -0.3730 |
|  | *Salix sphenophylla* | Deciduous | Low | 4 | -0.7263 | -0.7177 | -0.3909 | . |
|  | *Salix udensis* | Deciduous | Tall | 7 | -0.9042 | -0.8814 | -0.6096 | -0.7983 |
|  | *Vaccinium uliginosum* ssp. *vulcanorum* | Deciduous | Low | 5 | 0.6741 | 0.6736 | . | . |
|  | COMMUNITY-WIDE | . | . | 12 | -0.6231 | -0.5842 | -0.6139 | -0.8270 |
| Cairngorms | *Betula pendula* | Deciduous | Tall | 5 | -0.1191 | -0.1079 | -0.6825 | -0.8199 |
|  | *Calluna vulgaris* | Evergreen | Low | 9 | 0 | 0 | . | . |
|  | *Empetrum nigrum* | Evergreen | Low | 6 | -0.1853 | -0.1853 | . | . |
|  | *Pinus sylvestris* | Evergreen | Tall | 7 | 0.5925 | 0.5923 | . | . |
|  | *Vaccinium vitis-idaea* | Evergreen | Low | 7 | -0.5881 | -0.5900 | . | . |
|  | *Vaccinium myrtillus* | Deciduous | Low | 11 | -0.6855 | -0.6844 | . | . |
|  | COMMUNITY-WIDE | . | . | 12 | -0.0826 | -0.0624 | -0.4797 | -0.8036 |
| Caucasus | COMMUNITY-WIDE | . | . | 8 | 0.2126 | 0.1575 | 0.3537 | 0.0139 |
| Troodos | *Capparis spinosa* | Deciduous | Low | 4 | 0.1318 | 0.1321 | . | . |
|  | *Cistus creticus* | Evergreen | Low | 4 | 0.8687 | 0.8668 | . | . |
|  | *Pinus nigra* ssp. *pallasiana* | Evergreen | Tall | 4 | -0.2772 | -0.2772 | . | . |
|  | *Rhus coriaria* | Deciduous | Tall | 4 | -0.8922 | -0.4216 | . | -0.3463 |
|  | COMMUNITY-WIDE | . | . | 11 | -0.5407 | -0.5852 | 0.3121 | 0.1169 |

Table S4. Sources of variation in community-wide losses of woody plant foliage to insects (SAS GLIMMIX procedure, type 3 sum of squares).

| Effect | Explanatory variable | Test statistics | *P* |
| --- | --- | --- | --- |
| Fixed | Region | *F*_5, 57_=3.29 | 0.0111 |
|  | Guild | *F*_2, 114_=294.62 | <.0001 |
|  | Elevation | *F*_1, 57_=13.53 | 0.0005 |
|  | Region × Guild | *F*_10, 114_=3.97 | 0.0001 |
|  | Region × Elevation | *F*_5, 57_=3.75 | 0.0053 |
|  | Guild × Elevation | *F*_2, 114_=4.81 | 0.0098 |
|  | Region × Guild × Elevation | *F*_10, 114_=4.22 | <.0001 |
| Random | Site (Region) | *χ*^2^_1_=10.05 | 0.0008 |

Table S5. Pearson correlation coefficients (*r*) between community-wide leaf functional traits and elevation within individual mountain ranges.

| Mountain range | Water content | | SLA | | Area | |
| --- | --- | --- | --- | --- | --- | --- |
|  | *r* | *P* | *r* | *P* | *r* | *P* |
| Alps | -0.352 | 0.29 | -0.241 | 0.43 | -0.619 | 0.02 |
| Altai | 0.704 | 0.0073 | -0.414 | 0.16 | -0.771 | 0.0020 |
| Avachinskij | 0.173 | 0.59 | -0.504 | 0.09 | -0.867 | 0.0003 |
| Cairngorms | -0.560 | 0.06 | 0.339 | 0.28 | -0.755 | 0.0046 |
| Caucasus | -0.101 | 0.81 | -0.351 | 0.39 | -0.562 | 0.15 |
| Troodos | 0.562 | 0.07 | 0.632 | 0.04 | -0.375 | 0.26 |

Table S6. Pearson correlation coefficients (*r*) between log_10_(x+0.01)-transformed total community-wide loss of leaf area to insects and leaf functional traits within individual mountain ranges.

| Mountain range | Water content | | SLA | | Area | |
| --- | --- | --- | --- | --- | --- | --- |
|  | *r* | *P* | *r* | *P* | *r* | *P* |
| Alps | -0.479 | 0.14 | 0.085 | 0.78 | 0.013 | 0.97 |
| Altai | -0.790 | 0.0013 | 0.123 | 0.69 | 0.609 | 0.03 |
| Avachinskij | 0.332 | 0.29 | 0.290 | 0.36 | 0.680 | 0.01 |
| Cairngorms | 0.240 | 0.45 | 0.017 | 0.96 | 0.177 | 0.58 |
| Caucasus | -0.022 | 0.96 | -0.010 | 0.98 | 0.280 | 0.50 |
| Troodos | -0.284 | 0.40 | -0.040 | 0.91 | 0.430 | 0.19 |
